# Reflectance Modeling for Real Snow Structures Using a Beam Tracing Model

**DOI:** 10.3390/s8053482

**Published:** 2008-05-26

**Authors:** Dominik Bänninger, Claude Saskia Bourgeois, Margret Matzl, Martin Schneebeli

**Affiliations:** 1 Institute of Environmental Geosciences, Department of Environmental Sciences, University of Basel, Bernoullistrasse 30, 4056 Basel, Switzerland; 2 Institute for Atmospheric and Climate Science, ETH Zürich, Universitätstrasse 16, 8092 Zürich, Switzerland; E-mail: bourgeois@meteotest.ch; 3 WSL Institute for Snow and Avalanche Research, SLF, Flüelastrasse 11, Davos Dorf, Switzerland; E-mail: schneebeli@slf.ch

**Keywords:** reflectance, radiative transfer, modeling, snow

## Abstract

It is important to understand reflective properties of snow, for example for remote sensing applications and for modeling of energy balances in snow packs. We present a method with which we can compare reflectance measurements and calculations for the same snow sample structures. Therefore, we first tomograph snow samples to acquire snow structure images (6 × 2 mm). Second, we calculated the sample reflectance by modeling the radiative transfer, using a beam tracing model. This model calculates the biconical reflectance (BR) derived from an arbitrary number of incident beams. The incident beams represent a diffuse light source. We applied our method to four different snow samples: Fresh snow, metamorphosed snow, depth hoar, and wet snow. The results show that (i) the calculated and measured reflectances agree well and (ii) the model produces different biconical reflectances for different snow types. The ratio of the structure to the wavelength is large. We estimated that the size parameter is larger than 50 in all cases we analyzed. Specific surface area of the snow samples explains most of the difference in radiance, but not the different biconical reflectance distributions. The presented method overcomes the limitations of common radiative transfer models which use idealized grain shapes such as spheres, plates, needles and hexagonal particles. With this method we could improve our understanding for changes in biconical reflectance distribution associated with changes in specific surface area.

## 1. Introduction

The radiative transfer properties of snow are highly relevant for estimating the energy balance [1, 2], for interpreting remote sensing data [3] and also for biological applications [4]. The variation of the broadband albedo of snow mainly depends on the effective optical diameter [5, 6], whereas the effect of different irregular snow micro-structures on reflectance is so far not well understood. Especially, the effect of anisotropic shapes as those in new snow or those formed due to strong temperature gradients are poorly investigated [7, 8].

Two opposite approaches are possible to calculate the radiative transfer properties of a snow sample, i.e. a medium with a complex geometry: (i) The snow structure is simplified to such a degree that the scattering of electromagnetic waves can be solved exactly by radiative transfer theory [6,9], (ii) the snow structure is described with a high accuracy at the expense of simplifying the physics of light scattering to be able to calculate the radiative transfer. It is the second approach we invest in this study. The motivation for this decision were the findings that the reflectance of snow in the near infrared (NIR) somehow depends on the specific surface area, a measure which is used to characterize snow structure [10, 11].

Models derived from radiative transfer theory describe light scattering often on the basis of the concept of equivalent sphere diameter [6, 12, 13]. This concept is a crude approximation of the real snow. More recent approaches aim at including more realistic structural information of real snow structure: The grain is approximated by dielectric films, plates, needles, prisms and hexagonal particles [14, 15, 16, 17, 18]. In the study of [17] a ray tracing approach was presented which calculates scattering properties of single particles having complex geometries. Therefore, geometric optics and the far-field diffraction approximation were applied. Ray tracing algorithms based on Monte Carlo technique are also used to describe radiative transfer [19]. Such approaches have the advantage that many different physical properties can easily be calculated. But the difficulty in Monte Carlo based ray tracing approaches is to determine the probabilities of the physical processes (e.g. diffraction, reflection, absorption) as well as the representation of the structure of a porous medium.

A typical problem in radiative transfer modeling is the validation of the calculated results with measured data. To overcome this gap we present in this study radiative transfer calculations at the same structure for which the reflectance is measured. To reach this goal we used micro-tomography to image the microstructure of snow samples [20, 21] and used this structural information to model the radiative transfer. We modeled the radiative transfer within the snow samples using the beam-tracing model (BTM) presented in [22]. This radiative transfer model we present here calculates coherent multiple scattering. The BTM was originally designed to model the radiative transfer in soils. Snow is a stronger scatterer and much lower absorber than soil. Thus, in case of snow the number of light beams which have to be processed is a couple of orders larger than in case of soil. To make the calculations feasible we implemented a snow extension module in the BTM.

The representation of three-dimensional snow structure and the beam tracing in three-dimensional space is expensive with respect to computer memory and computation time. Thus, the BTM was implemented to run in two-dimensional space. Reducing dimensionality from three to two dimensions causes loss of structured information. For strongly absorbing media it was shown that the differences between radiative transfer calculations for the two-dimensional and three-dimensional case are of minor importance [23]. Since snow is a low absorbing medium the light can easily penetrate through the grains and does not have to follow the pore space. Thus we can expect that reduction from three- to two-dimensional case is even less important for snow than for strongly absorbing media. We will demonstrate that two-dimensional cross sectional images of snow are already sufficient for obtaining representative results.

## 2. The beam tracing model (BTM)

The principle of our radiative transfer model is to take a structure, to illuminate the structure with beams and to follow each beam until it is absorbed or leaving the sample. To do this, we have to describe the structure and then to calculate the optical paths of the beams. Because this procedure is numerically expensive we had to simplify the physics to calculate the optical path of the beam by neglecting the phenomena of diffraction. This simplification is legitimate by the fact that the resolution of the structures is large compared to the wavelength and thus the actual cross section is close to the scattering cross section of the particle [24, 25]. Even for the finest structures we calculated the size parameter to be *x* > 50. The size parameter is defined as *x* = 2*πa* / *λ* with *a* being the radius of the sphere and *λ* the wavelength. In the following sections the technical implementations of the model are explained in detail.

### 2.1 Morphology of the scattering medium

The BTM requires a detailed description of the structure of the scattering medium. The input format of this information is given in the form of a pixel image. We call this image the structure image. Cross sections through tomographed snow cubes are used to retrieve the structure images. The resolution of the structure images depends on the scan resolution of the tomograph. The structure image is a binary image representing the spatial distribution of the ice and air phase. To each of the two phases we assign a complex refractive index to describe its optical properties. Before running the BTM we preprocessed each structure image by detecting the contour line pixels between the air and ice phase and by determining its local orientation. The later is done by determining the normal to the contour line at the respective pixel. This information is required to calculate the optical path of a light beam (details of the algorithm are given in [22]).

**Figure 1.**
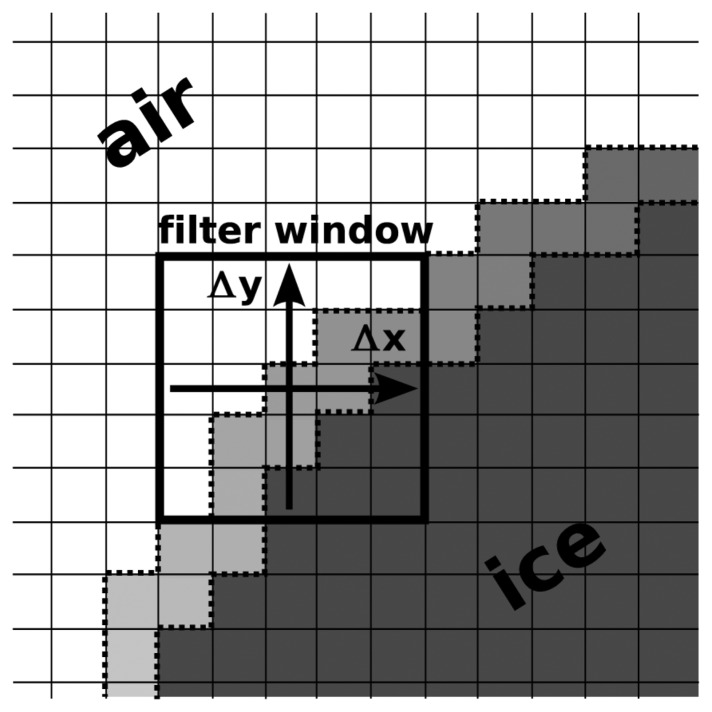
Typical elements of a structure image shown at a cutout of an air/ice interface. The white pixels represent the air phase and the dark gray pixels represent the ice phase. The different gray values of the contour line – the contour line pixels are highlighted by the dotted lines – represent the local orientation of the air/ice interface.

To calculate the local orientation of the contour line pixels we make use of image analysis techniques. A contour line pixel is in our context an ice pixel that has at least one of its eight neighbor pixels being an air pixel. We calculated the local orientation of a contour line pixel by determining the x- and y-derivative of the pixel color value at the position of the contour line pixel. The derivatives are calculated with the edge detection filter presented in [26]. This edge detection filter works with a window size of 5×5 pixels (Figure 1) Using the law of Pythagoras the normal to the contour line pixel is derived from the x- and y- derivatives. We assigned this value to the contour line pixel. This algorithm is applied for each contour line pixel so that finally each boundary pixel of the structure contains its local orientation.

### 2.2 Illumination of the sample

The BTM illuminates the structure image at the top edge with a user defined number of beams. An incident beam is defined by its direction, position, and intensity. The illumination can be diffuse, collimated, or partially diffuse. We used 200 incident beams for each simulation run. The incident beams were randomly distributed with an incident angle of ± 60° around the zenith. This approximates the illumination of the Ulbrichts sphere which was used in the experiment.

The BTM can be run at any wavelength as long as the refractive indices are known and the geometric optic is valid. We ran the model at the wavelength of 870 nm. We selected this wavelength because at shorter wavelengths the influence of soot gets more significant, and thus corrupts the comparison with measured reflectance. The complex refractive index of the snow at this wavelength is *nˆ*_*λ*=870_ = 1.303 + *i*4.34·10^−7^ [27, 28].

### 2.3 Beam tracing

The radiative transfer is calculated by tracing the path of light beams pixel-by-pixel through the structure image. The optical path of a beam is defined by its position and propagation direction and the subsequent scattering events, calculated with Snell's Law and the Fresnel equations. According to the Fresnel equation, a light beam scattered at an optical interface is split into transmitted and reflected part. The continuous splitting of the beams by scattering yields a large number of beams which have to be processed. Therefore, this algorithm becomes computationally demanding.

At the left and right edge of the sample image we used completely reflective mirrors simulating an infinitely wide sample. This assumption reduces boundary effects caused by the limited size of the sample. Light beams reaching the bottom of the image are assumed to leave the snow sample and are accounted for as transmitted light. The intensities of these beams were stored because this value is needed in the snow extension module, described in the following section. The propagation directions and intensities of the light beams leaving the sample at the upper edge were stored as well. This yields the biconical reflectance (BR) [29]. The hole algorithm of the BTM is described in detail in [22].

### 2.4 Snow extension module for BTM – the ladder approximation

The BTM was developed to calculate the radiative transfer in soil samples. In the case of soil, the light is absorbed within the first millimeters of the sample. In case of snow, the penetration depth of light is a couple of orders larger. Consequently, the number of light beams which have to be processed increases drastically.

**Figure 2.**
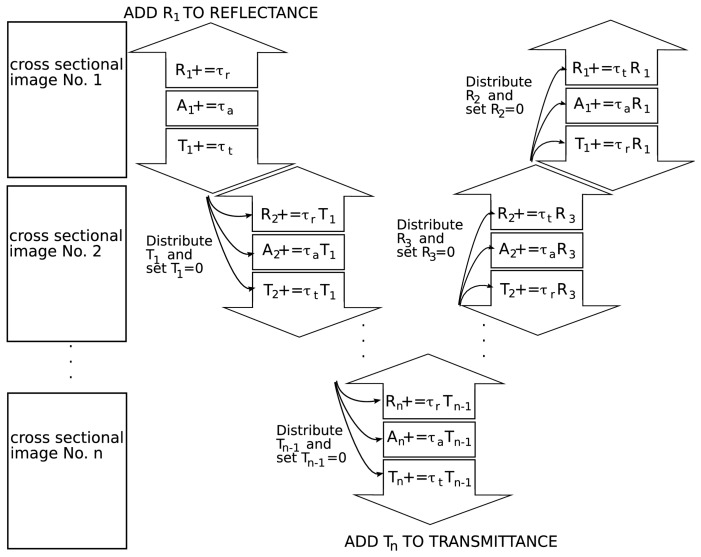
Calculating the reflectance of a snow colon by piling up several small cross sectional images. *τ_r_*, *τ_a_*, and *τ_t_* are the reflectance, absorption, and transmittance coefficients; R, A and T are the cumulative reflectance, absorption, and transmittance. The notation “X+=…” stands for “X=X+ ….”.

To solve this problem we reduced the computational demand by running the beam tracing model only for a small cross sectional image. The size of the image was determined by the scan range of the tomograph but should at least contain multiple snow grains. With the BTM we calculated the reflectance *τ_r_*, absorption *τ_a_* and transmittance *τ_t_* for these cross sectional images. To extrapolate the radiative transfer from the small cross sectional images to a snow column we virtually multiply a structure image and pile them up. Since each of the multiplied structure images has the same radiative transfer properties, the reflectance of the snow column can be calculated with the iterative algorithm outlined in Figure 2. This procedure is also called the ladder approximation. The number of slices *n* used to build up a snow column was selected in such a way that the transmission through the snow column is close to zero and the reflectance does not change anymore when additional slices would be added at the bottom of the snow column. In this study we set *n* =100. The iterative algorithm was repeated until the reflectance of the snow column converges to a constant value. For our cases 100 iterations were enough to get stable results.

### 2.5 Sensor

**Figure 3.**
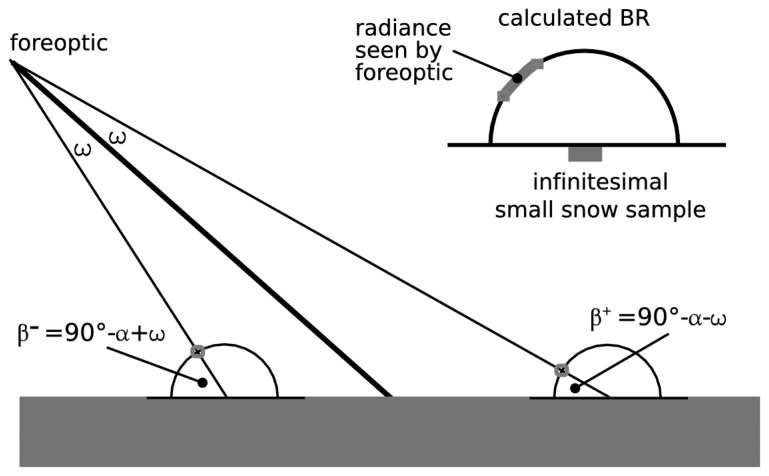
Calculating the reflectance seen by the sensors fore optic. The half opening angle of our fore optic was *ω* = 1.5°. The angle of 90°-*α* corresponds to the viewing angle which is in our setup equal to 68°. The little sketch in the upper right corner illustrates which range of the BR is seen by the fore optic when being far away from the snow sample.

The reflected light seen by the sensor is defined by the field-of-view given by the sensors fore-optic. The reflectance is computed from the BR as outlined in Figure 3 [30]. In case the field of view of the sensor is small, the recorded radiance depends strongly on the sensors position and on the shape of the BR. Since we can compute only a small, finite number of incident beams we get a noisy BR which is disadvantageous for a sensor with small field of view. This problem can be reduced when having a smoother BR. To get a smoother BR we simultaneously apply three different approaches: (i) For each sample we run the model several times and calculate the mean BR of the single model runs. Repeatedly running the model with a given number of incident beams (with a randomized distribution) is equivalent with running the model once with a multiple number of incident beams. (ii) For each angle of the BR we calculate the mean value from the reflectance at the angles with the same negative and positive angle. We are allowed to do this because the incidence of the axial symmetric Ulbricht sphere is normal, thus the BR is axial symmetric with respect to the normal of the sample surface. (iii) For further smoothing of the BR we applied a moving average.

## 3. Experiment

### 3.1 Snow sampling

We collected four samples of different snow types with the dimension of approximatively 30 x 30 x 30 cm to measure the reflectance. We classified the snow samples as fresh snow, metamorphosed snow, depth hoar and wet snow. The sampling site was in Davos, Switzerland (February 2006). The snow samples were stored separately in Styrofoam boxes for transportation to the nearby cold laboratory. The following measurements (reflectance measurements and tomography) were done in the cold laboratory quickly after each other to avoid that snow metamorphosis alters the structure significantly.

### 3.2 Snow reflectance measurement

**Figure 4.**
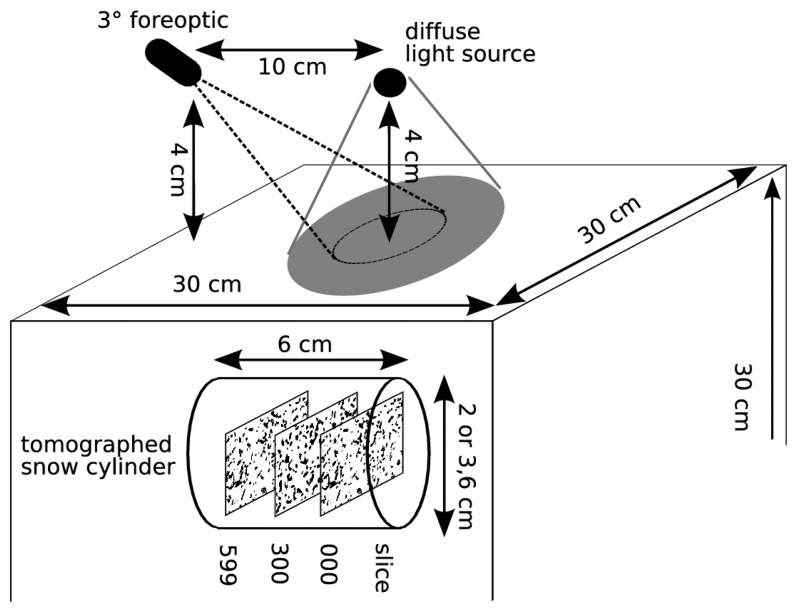
Experimental setup for measuring the reflectance of a snow sample. The description above the snow cube describes the reflectance measurement. The cylinder within the snow cube illustrates that we extracted after the reflectance measurement a snow sample of 6cm height. From this cylinder we tomographed a snow layer resulting in 600 tomographed slices. These cross sections are then used as input for the beam tracing model.

For the reflectance measurement in the laboratory we took the snow cube out of the Styrofoam box and prepared the sample surface with a sharp metal plate to be completely flat. The reflectance of the samples was measured with a standard field spectrometer “Field Spec Pro Dual VNIR” from “Analytical Spectral Devices”. The spectrometer measures the spectrum from 350 to 1050 nm using a 512-channel silicon photo-diode array. Since reflectance of snow varies strongly in the near infrared (NIR) spectrum [6] we made our analysis for one single wavelength, *λ*=870 nm. For our radiance measurements we attached a 3° field-of-view fore-optic to the glass fiber. Each sample was scanned 15 times (five repetitions at three different positions at the snow surface) to minimize measuring errors. The three positions were always close to the center of the surface of the snow sample. The geometric arrangement of the light source and the detector, and the distance of them to the snow surface were kept constant with respect to measured position at the snow surface (Figure 4).

The snow sample was illuminated with a quartz lamp mounted inside an Ulbricht sphere which produces light which is nearly 100% diffuse. Since the Ulbricht sphere was positioned a few centimeters above the sample, the sample surface illumination was conical. The samples were illuminated with the entire wavelength spectrum of the light source. Before the reflectance of each sample was measured, the reflectance of a white reflecting reference (Spectralon panel, Labsphere) was measured. The reflectance was calculated according to the relation *R_λ_* = *L_λ,snow_* / *L_λ,spectralon_* where *R_λ_* denotes the spectral reflectance and *L_λ_* the measured radiance for snow and Spectralon, respectively. The experimental setup for measuring the reflectance is depicted in Figure 4.

### 3.3 Snow tomography

For each snow sample we extracted one horizontal cylinder with a length of 6 cm and a diameter of 2 or 3.6 cm. These cylindrical samples were tomographed in a X-ray micro-tomograph (Scanco micro-CT 80). The scan resolution was adapted to the structural size of the samples in such a way that the smallest structure was always at least 2 to 4 times larger than the size of the voxel [31]. Thus, the resulting voxel size of the tomographed structure was 10 *μ* m (fresh snow, metamorphosed snow) and 18 *μ* m (depth hoar, wet snow) [31]. The resolution of the tomography is directly used for the structure images. Thus, the pixel size in the structure images is 10 *μ* m and 18 *μ* m, respectively.

We used the specific surface area (SSA) as structural parameter to describe the snow structure. The SSA has been used for several years as an optical equivalent sphere to describe optical properties of general polydispersions [33] and snow [13]. This property has also been used to parameterize structures for radiative transfer modeling of snow [34]. Alongside its use for the optical description of snow, the SSA is an important parameter for describing the structural size and the geometry of sintered media. We measured the SSA using the triangulated surface of the volume data [35]. In the following the SSA is defined as surface-to-volume ratio [mm^-1^].

## 4. Results

### 4.1 Results from measurements

We tested the homogeneity of the collected snow with a high-resolution snow penetrometer [36], both horizontally and vertically. The variation of the penetration hardness was about 15% for all samples. From this result we conclude that each snow sample is homogeneous so that we can compare the reflectance measurement taking at the top of the snow sample with the results calculated from the cylindrical tomographed sample.

**Figure 5.**
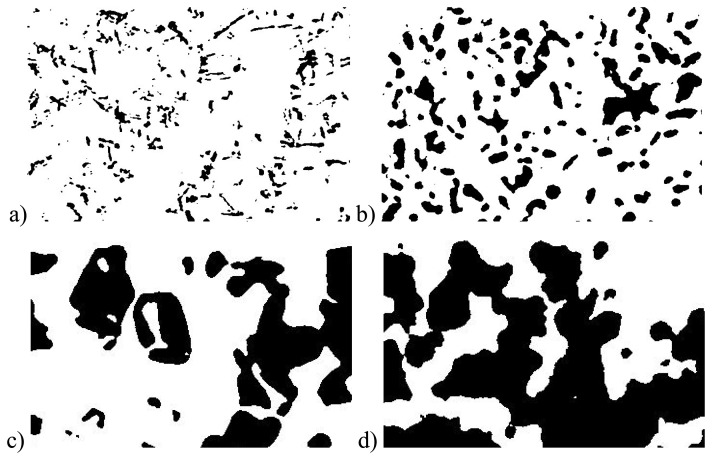
Tomographed cross sections of the snow samples. White depicts the air phase, black the ice. The cross sections are: a) Fresh snow, b) metamorphosed snow, c) depth hoar, and d) wet snow. The dimension of the images is 6 × 4 mm.

**Figure 6.**
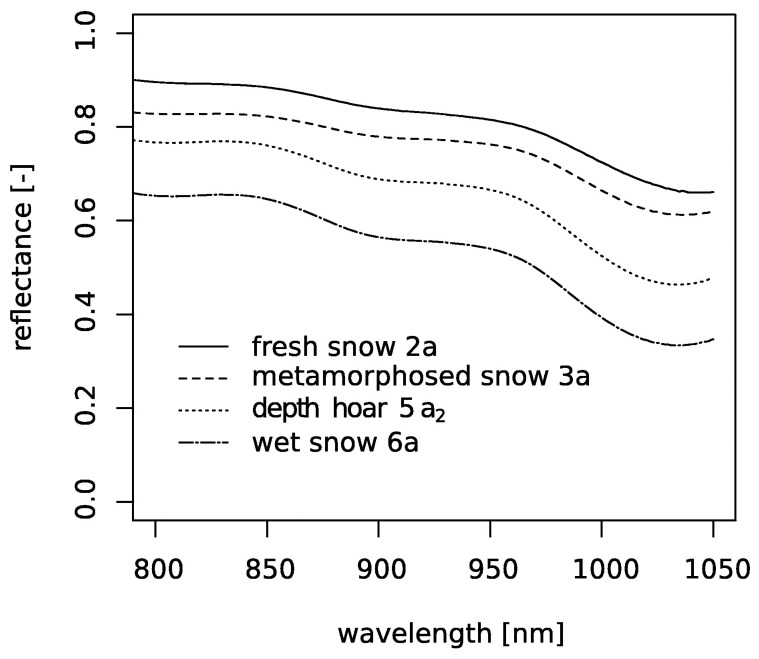
Measured reflectance spectra of the snow samples. The abbreviation behind the snow name is the class according to the international snow classification.

Table 1 summarizes the characteristics of the four snow samples which were collected in February 2006 in the area of Davos, Switzerland. Figure 5 shows for each snow sample a structure image recorded with the tomograph. In Figure 6 we plotted the measured reflectance spectra of these samples.

### 4.2 Results from modeling

**Figure 7.**
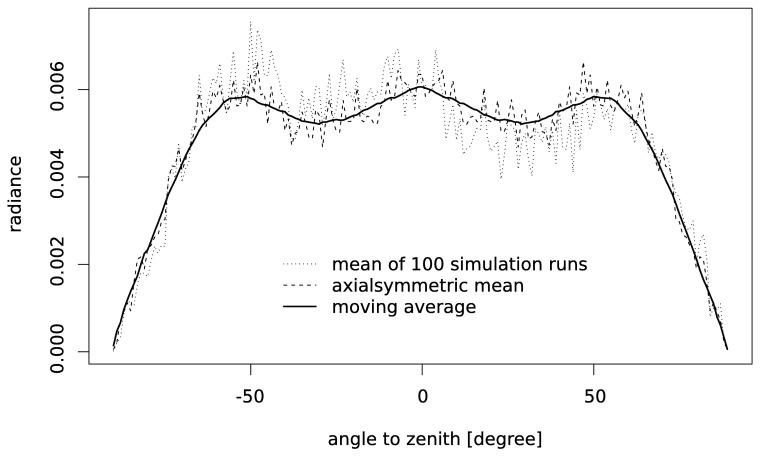
Calculated radiance for the first structure image of the fresh snow. The dotted line indicates the mean BR for the 100 simulation runs. The dashed line is the mean of the radiances for the angle with the same absolute value of negative and positive angles. The solid line represents the dashed line where we applied a moving average filter with a window width of 10 degree.

**Figure 8.**
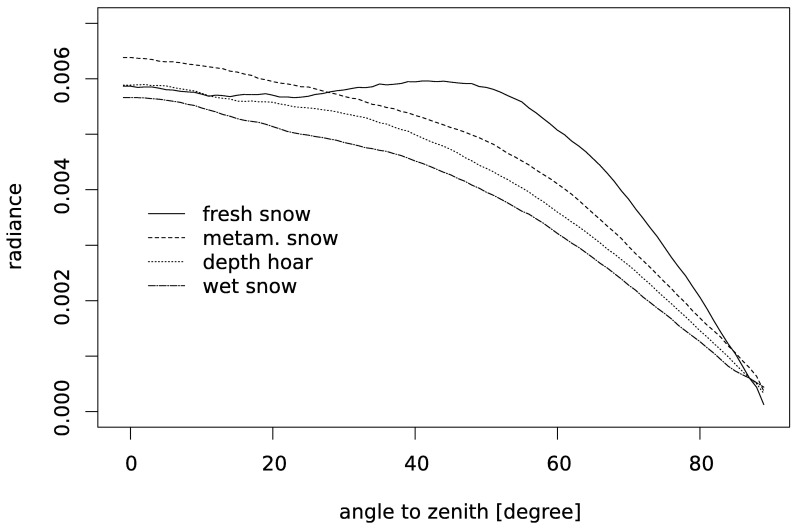
Calculated radiance for each viewing angle in the upper hemisphere.

For each of the four snow samples we selected three different cross sections as structure images, the 0th, 300th and 599th pixel layer from the tomographed cylinder (Figure 4). Taking three different structure images allows to test for the effect of the variability of the snow structure within the tomographed sample. To get a realistic representation of the light incidence we used 200 incident beams for each run. For each structure image we started 100 model runs and calculated the mean to get the angle dependent radiance (Figure 7). The resulting BR is slightly asymmetric and noisy. Thus, we calculated next the mean of the radiances for the angle with the same absolute value of negative and positive angles. For this BR we applied additionally a moving average filter with a window width of 10 degree resulting in a smooth, symmetric BR (Figure 8).

**Figure 9.**
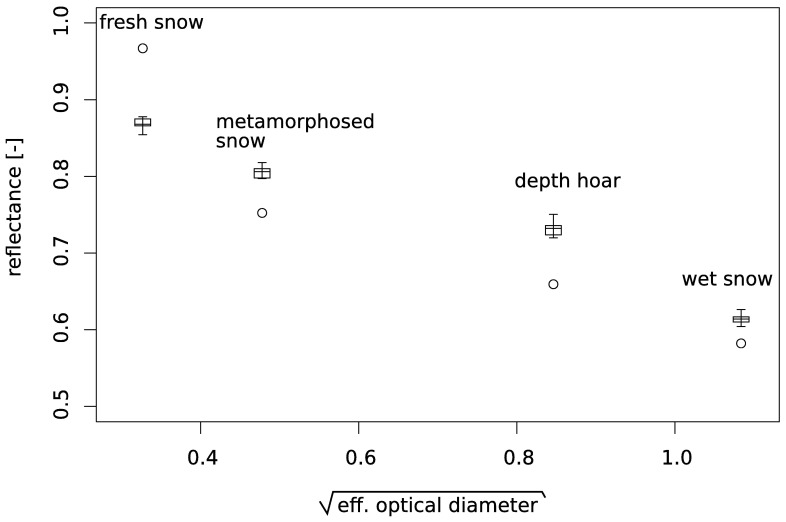
Measured (boxplots, showing maxima, minima, and quartiles) and calculated reflectance (circles) plotted against the square root of the effective optical diameter. These results are calculated by taking the radiance at 68° from Fig. 8.

The calculated reflectance against the effective optical diameter $\sqrt{D}$ calculated with the relation [37, 6]

(1) $$D=\frac{6}{\text{SSA}}$$

is shown in Figure 9. Here, we compare how well the computed reflectances fit the measured reflectances.

## 5. Discussion

It was reported for wavelengths shorter than 1000 nm that the measured and calculated reflectances follow a linear trend between the reflectance and the square root of the effective grain diameter [6] (Figure 9). For our data we found a good agreement to this statement: The squared linear regression coefficient is R^2^=0.96 for the reflectance measurement and R^2^=0.86 for the calculated reflectances. This linear relationship is valid for wavelengths shorter than 1000 nm.

From Figure 9 we found that the model overestimates the reflectance for fresh snow and underestimates the reflectance for the other three samples. Possible sources yielding differences between measurements and calculations are: The measured snow surface is different from the imaged snow cube, the preparation of the snow surface before measurement disturbs snow structure, the area of structure images are too small to reflect a representative area. The disturbance of the snow structure by surface preparation might be especially significant for the fresh snow (most fragile structure) which could explain the overestimation of the reflectance by the model. Since we neglect diffraction, as outlined in the description of the model, some differences between measurement and computed results will differ due to this simplification of the model. The effect of diffraction will be more pronounced for the gracefully build fresh snow and continuously decrease for coarser structures [17, 12].

From the simulated BR we find that the change of the radiance with the angle is very sensitive above an absolute angle of 60°. Changing the viewing angle from 68° to 70° for fresh snow or from 68° to 66° for metamorphosed snow would yield correct calculated reflectances. Thus, inaccurate installation of the sensors height above the sample of ± 4 mm would be enough to get discrepancies as shown in Figure 9. Since we were not aware about this high sensitivity during the experiment it is possible that some part of the error must be explained by measurement inaccuracies.

From the single simulation runs we found that 200 incident beams are not sufficient to get a BR with low noise. Obviously, the single channels of the BR are far-away from convergence (cf. Figure 7). For the overall reflectance we found that the changes of the reflectance converges with each additional beam (from the first to the 200th beam) to a constant value. It was reported that radiative transfer problems require a number of photons in the order of 10^7^ to solve a radiative transfer problem accurately for a low absorbing medium [38]. When we sum up the number of scattering events caused by our 200 incident beams we get a total number between 10^6^ and 10^7^ which would explain why we observe convergence for the overall reflectance.

Comparing the results of the different structure images taken from the same sample we found different calculated reflectances. This variance is caused by the comparison of images, which contain different structural information, and the random distribution of the 200 incident beams. Nevertheless, the variance in computed reflectances is approximately within the range of measurement variance. Thus, even when we consider only two-dimensional structure information we find that the presented method is very efficient opposing the computation demand to the accuracy of the results.

## 6. Conclusions

With this study we could demonstrate by the direct comparison of reflectance measurements and radiative transfer modeling for the same snow structure that the modeling approach is very promising. With the BTM we presented a new approach to simulate the reflectance for natural snow structures. This model approach closes the knowledge gap of predicting the biconical reflectance for given snow structures. We found a good general agreement between the measured and simulated reflectance. We performed the radiative transfer experiments and calculations at a wavelength of 870 nm because at this wavelength the influence of soot and light absorption by fluid water is minimal.

From the results we found that the BTM overestimates the reflectance for fresh snow and underestimates the reflectance for snow samples with coarser structure. Under the assumption that the reflectance measurements are correct the deviations can be explained by not considering the process of refraction and by the fact that we do not know the size of the representative areas of the structure images. The representative area might be larger than the images used in this study, the asymmetric BR is an indication for this. However, we think that this method could be used in the future to calculate the bidirectional reflectance distribution function (BRDF) of different, and especially layered, snow types. It remains to discuss whether modeling radiative transfer in three-dimensional space improves the modeling results.

## Figures and Tables

**Table 1.** Description of the snow samples. ISC means international snow classification, the image size is the size of the cross-sectional images used for simulating radiative transfer with the BTM.

Snow type (ISC)	Shortcut	density [kg/m^3^]	SSA [mm^-1^]	effective radius *μ* m	cross sectional image size [mm × mm]
Fresh snow (2a)	fs	110	59.22	51	6 × 4
metam. snow (3a)	m2	194	26.29	114	6 × 4
depth hoar (5a_2_)	dh	305	8,38	358	10.8 × 7.2
wet snow (6a)	ws	535	5.11	587	10.8 × 7.2
